# Revisiting the *Trypanosoma cruzi* metacyclogenesis: morphological and ultrastructural analyses during cell differentiation

**DOI:** 10.1186/s13071-018-2664-4

**Published:** 2018-02-06

**Authors:** Camila Silva Gonçalves, Andrea Rodrigues Ávila, Wanderley de Souza, Maria Cristina M. Motta, Danielle Pereira Cavalcanti

**Affiliations:** 1 Laboratório de Ultraestrutura Celular Hertha Meyer, Instituto de Biofísica Carlos Chagas Filho, UFRJ, Rio de Janeiro, RJ Brazil; 2 Laboratório de Microbiologia, Diretoria de Metrologia Aplicada às Ciências da Vida, Instituto Nacional de Metrologia, Qualidade e Tecnologia- Inmetro, Rio de Janeiro, RJ Brazil; 3 Laboratório de Regulação da Expressão Gênica, Instituto Carlos Chagas, FIOCRUZ, Curitiba, PR Brazil; 4 Instituto Nacional de Ciência e Tecnologia de Biologia Estrutural e Bioimagem, Universidade Federal do Rio de Janeiro, Rio de Janeiro, RJ Brazil

**Keywords:** Cell differentiation, 3D reconstruction, kDNA arrangement, Metacyclogenesis, Microscopy techniques, *Trypanosoma cruzi*, Ultrastructure

## Abstract

**Background:**

*Trypanosoma cruzi* uses several strategies to survive in different hosts. A key step in the life-cycle of this parasite is metacyclogenesis, which involves various morphological, biochemical, and genetic changes that induce the differentiation of non-pathogenic epimastigotes into pathogenic metacyclic trypomastigotes. During metacyclogenesis, *T. cruzi* displays distinct morphologies and ultrastructural features, which have not been fully characterized.

**Results:**

We performed a temporal description of metacyclogenesis using different microscopy techniques that resulted in the identification of three intermediate forms of *T. cruzi*: intermediates I, II and III. Such classification was based on morphological and ultrastructural aspects as the location of the kinetoplast in relation to the nucleus, kinetoplast shape and kDNA topology. Furthermore, we suggested that metacyclic trypomastigotes derived from intermediate forms that had already detached from the substrate. We also found that changes in the kinetoplast morphology and kDNA arrangement occurred only after the repositioning of this structure toward the posterior region of the cell body. These changes occurred during the later stages of differentiation. In contrast, changes in the nucleus shape began as soon as metacyclogenesis was initiated, while changes in nuclear ultrastructure, such as the loss of the nucleolus, were only observed during later stages of differentiation. Finally, we found that kDNA networks of distinct *T. cruzi* forms present different patterns of DNA topology.

**Conclusions:**

Our study of *T. cruzi* metacyclogenesis revealed important aspects of the morphology and ultrastructure of this intriguing cell differentiation process. This research expands our understanding of this parasite’s fascinating life-cycle. It also highlights the study of *T. cruzi* as an important and exciting model system for investigating diverse aspects of cellular, molecular, and evolutionary biology.

## Background

*Trypanosoma cruzi* is the parasite that causes Chagas disease, a neglected parasitic infection that affects an estimated 8 million people worldwide, mainly in Latin America [1]. In endemic areas, the most common mechanism for *T. cruzi* infection is through the bite of triatomine insects. However, Chagas disease can also be transmitted through organ transplants, blood transfusions, contaminated food and from mother to child. *Trypanosoma cruzi* is a heteroxenic protozoan, which means that it presents different stages of its life-cycle in distinct hosts. Since it inhabits both invertebrate and vertebrate hosts, this microorganism uses several strategies to survive in these diverse environmental conditions [2]. During its life-cycle, the parasite undergoes changes in morphology, metabolism, and gene expression as it passes from the epimastigote replicative stage in the insect to the metacyclic trypomastigote form, which infects humans. Epimastigotes and amastigotes are the replicative forms found in the insect midgut and mammalian cells, respectively. Meanwhile, the metacyclic trypomastigote (found in the insect vector) and the bloodstream trypomastigote (present in the vertebrate host) are the non-proliferative and infective stages. In addition to these distinct and well-studied stages, intermediate forms are also observed during the *T. cruzi* life-cycle (reviewed in [3, 4]). For these reasons, *T. cruzi* is an excellent model for studying diverse aspects of cell differentiation. Cell differentiation in this parasite is triggered by environmental factors, such as starvation, pH and temperature changes. This allows the protozoan to successfully adapt to the environment and efficiently colonize a host [5].

The differentiation of epimastigotes into metacyclic trypomastigotes, a process known as metacyclogenesis, occurs in the midgut of the vector organism and is a fundamental step in the life-cycle of *T. cruzi*. During metacyclogenesis, bloodstream trypomastigotes differentiate into replicative epimastigotes in the host insect’s stomach, which divide in the midgut and subsequently migrate to the insect’s rectum. There, each epimastigote adheres to the epithelium via a flagellum prior to differentiation into a non-replicative, infective metacyclic trypomastigote. These infective forms are then released into the excreta of the triatomine insect while it feeds on the blood of a vertebrate [6, 7]. The factors that trigger metacyclogenesis are still unknown, but previous studies reported that this transformation might be stimulated by nutritional starvation, cyclic AMP, and adenylate cyclase [8].

Metacyclogenesis can be reproduced in vitro under chemically defined conditions. To induce differentiation, epimastigotes are submitted to nutritional stress by a short incubation in an artificial medium (TAU medium) that has a composition and pH similar to triatomine urine, followed by a longer incubation in TAU medium supplemented with amino acids and glucose (TAU3AAG) [9]. Similar to what occurs in the insect vector, epimastigotes in the differentiation medium adhere to the culture flask and then differentiate into metacyclic trypomastigotes. One advantage of this in vitro methodology is that parasites at several stages of development can be readily obtained. This facilitates the study of biological properties of both differentiated and intermediate forms of *T. cruzi*. Several studies have demonstrated that biochemical and genetic changes precede the morphological changes associated with parasite differentiation, which have been corroborated by proteomic analyses [10–13]. Furthermore, *T. cruzi* derived from different subgroups, also referred as Discrete Typing Units (DTUs) that harbor polymorphisms and display distinct morphologies during metacyclogenesis, indicating that the genetic diversity of this parasite might influence its ability to evolve and disperse to new hosts in nature [14, 15].

The phenotypic changes that occur in the parasite during metacyclogenesis include nuclear shape alterations, chromatin remodeling, a decrease in reservosome volume, an increase in the relative volumes of the kinetoplast and lipid bodies, and mitochondrial DNA rearrangement [16, 17]. The kinetoplast harbors the mitochondrial DNA (kDNA) of trypanosomatids and presents an unusual arragement formed by catenated circular molecules. Epimastigotes and amastigotes of *T. cruzi* possess a disk-shaped kinetoplast containing densely packed kDNA fibers, while trypomastigotes exhibit a globular structure filled with loosely arranged kDNA [16, 18]. Kinetoplast-associated proteins likely mediate the topological changes that kDNA undergoes during metacyclogenesis [19].

Although morphological and ultrastructural changes occurring in the nucleus and kinetoplast of *T. cruzi* during metacyclogenesis have been previously reported, the intermediate forms were poorly characterized, thus making its classification confusing and controversial. In this work, we performed a detailed structural characterization of *T. cruzi* during metacyclogenesis using numerous microscopy techniques, including scanning and transmission electron microscopy (SEM and TEM, respectively), focused-ion-beam scanning electron microscopy (FIB-SEM), and atomic force microscopy (AFM). These last two approaches generated three-dimensional (3D) models of the parasite during differentiation, and information about changes in kDNA topology.

Since metacyclogenesis represents an important model to study cell differentiation and parasite evolution, we re-visited the ultrastructural changes that occur during this process in *T. cruzi* considering: the repositioning of the kinetoplast and flagellum in relation to the nucleus, changes in the kinetoplast and cell body morphologies, as well as nuclear architecture and kDNA arrangement. As a result of this original and meticulous morphological analysis we present here a temporal description of metacyclogenesis, where intermediate forms were identified and may serve for future biochemical and molecular analyses.

## Methods

### Cell culture and in vitro metacyclogenesis

*Trypanosoma cruzi* epimastigotes (Dm28c clone) were cultivated in liver infusion tryptose (LIT) medium [20] supplemented with 10% fetal calf serum at 28 °C for 72 h. For in vitro differentiation, epimastigotes were grown until they reached a density of 5 × 10^7^ cells/ml. They were allowed to differentiate under chemically defined conditions as previously described by [9]. Briefly, epimastigotes were harvested from the LIT medium by centrifugation at 5000×*g* for 15 min at 10 °C and submitted to nutritional stress by incubation for 2 h in TAU medium [190 mM NaCl, 17 mM KCl, 2 mM MgCl_2_, 2 mM CaCl_2_, and 8 mM sodium phosphate buffer (pH 6.0)] at a density of 5 × 10^8^ cells/ml. The parasites were then diluted 100-fold in TAU3AAG medium (TAU medium supplemented with 50 mM sodium glutamate, 10 mM L-proline, 2 mM sodium aspartate, and 10 mM glucose) and allowed to attach to the culture flasks at 28 °C for 72 h. Metacyclic trypomastigotes were purified from the culture supernatant by ion exchange chromatography using 2-(diethylamino) ethyl ether cellulose (DEAE-cellulose) columns [21]. To obtain adhered intermediate forms, the attached parasites were collected 24, 48 and 72 h after nutritional stress by discarding the supernatant and vigorously shaking the samples in TAU3AAG medium at room temperature. The process was monitored by observation on an inverted microscope to ensure that the parasites had detached from the flask and then the sample was collected for further analysis. Supernatants were also collected after 48 and 72 h of differentiation. Three independent experiments were carried out on different days using different cell batches.

### Fluorescence microscopy

Protozoa were collected by centrifugation at 2000×*g*, washed once with PBS (phosphate buffered saline) pH 7.4, fixed in 4% paraformaldehyde in the same solution, mounted on poly-L-lysine-coated microscope coverslips (20 × 20 mm) and permeabilized using 1% Triton X-100 in PBS for 10 min. Slides were incubated for 30 min in blocking solution (3% bovine serum albumin, 0.5% fish gelatin and 0.02% Tween 20 in PBS) and then incubated for 1 h with the monoclonal antibody mAb2F6 diluted 1:10 in the blocking solution, at room temperature. Slides were washed with PBS, incubated with Alexa-Fluor 546-conjugated anti-mouse IgG for 1 h, washed with PBS and incubated with 10 μg/ml 4′,6-diamidino- 2-phenylindole (DAPI, from Molecular Probes, Oregon, USA) for 10 min. The slides were washed with PBS, mounted using ProLong Gold (Molecular Probes), and visualized using the Axio Observer (Zeiss, Jena, Germany) light microscope. The monoclonal antibody 2F6 (mAb2F6), which recognizes a paraflagellar rod protein, was kindly provided by Dr. Sergio Schenkman, Unifesp Brazil.

### Scanning electron microscopy

Sample processing was carried out using glass coverslips pre-coated with 1 mg/ml poly-L-lysine. Parasites were fixed for 1 h in 2.5% glutaraldehyde diluted in cacodylate buffer [0.1 M (pH 7.2)]. Cells were then adhered to coverslips, post-fixed for 1 h with 1% osmium tetroxide diluted in cacodylate buffer, and dehydrated in a graded alcohol series (50%, 70%, 90%, and two exchanges of 100% ethanol for 10 min each step). Samples were critical-point dried in a Leica EM CPD030 apparatus (Leica, Wetzlar, Germany). Specimens were coated with platinum in a Leica EM SCD050 before visualization using a Zeiss EVO 40 VP scanning electron microscope.

### Transmission electron microscopy

Protozoa were fixed for 1 h in 2.5% type II glutaraldehyde (Sigma, Missouri, USA) diluted in 0.1 M cacodylate buffer (pH 7.2). They were washed twice in cacodylate buffer and post-fixed (1% osmium tetroxide, 0.8% potassium ferrocyanide, 5 mM calcium chloride diluted in 0.1 M cacodylate buffer) for 1 h. Samples were then washed in cacodylate buffer, dehydrated in a graded series of acetone solutions (50%, 70%, 90%, and two exchanges of 100% acetone) for 10 min each step, and embedded in Polybed resin. Ultrathin sections were stained with 5% uranyl acetate for 45 min and lead citrate for 5 min before observation in a TECNAI Spirit (FEI) TEM operating at 80 kV.

### Focused-ion-beam scanning electron microscope

Samples were mounted on a support, coated with a thick carbon or gold layer, and imaged using a Hellios 650 microscope (FEI Company, Tokyo, Japan) or Auriga 45–38 (Zeiss) equipped with a gallium ion source for focused ion beam milling, a field emission gun, and an in-lens secondary electron detector for SEM imaging. To create a slice-and-view image series, a step size of 30 nm was chosen to remove material from the specimen surface with the focused ion beam. After image capturing, the back-scattered electron images had their contrast inverted to look like conventional TEM images. The slice-and-view series was automatically aligned using IMOD algorithms, and then a fine alignment was performed using MIDAS. Three-dimensional reconstruction models and measurements were performed using 3DMOD [22].

### Isolation of kDNA

The kDNA from the epimastigotes, intermediate forms, and trypomastigotes was isolated using a modified protocol based on [23]. Briefly, the protozoa (5 × 10^7^ cells/ml) were harvested by centrifugation and washed in NET 100 buffer [10 mM Tris-HCl (pH 8.0), 100 mM NaCl, and 100 mM EDTA]. The cells were then lysed at 37 °C for 60 min in 630 μl of NET 100 buffer containing 56.5 μl of 20% SDS and 11 μl of proteinase K (20 mg/ml). The lysate was loaded onto a 20% sucrose cushion and centrifuged for 20 min at 18,000×*g* in an Eppendorf 5810R centrifuge (Eppendorf, Hamburg, Germany). The kDNA pellet was resuspended in NET 100, loaded onto a second sucrose cushion, and processed as previously described. The kDNA was dialyzed against Tris-EDTA buffer [10 mM Tris-HCl (pH 8.00) and 1 mM EDTA] overnight, centrifuged at 18,000× *g* for 30 min, and resuspended in 1 mM Tris-HCl (pH 8.0).

### Atomic force microscopy (AFM)

The isolated networks of kDNA were prepared for AFM as described by [24]. Briefly, a solution containing the networks of kDNA (50–100 ng/ml) was mixed with 25 mM MgCl_2_ for 1 min and then deposited onto a freshly cleaved mica surface. The samples were washed with Milli-Q water, followed by a 1:1 ethanol:water mixture and then 100% ethanol. Samples were critical-point dried in a Leica EM CPD030 apparatus. The images of kDNA networks were acquired with a Bioscope Catalyst atomic force microscope (Bruker, Santa Barbara, CA, USA) in air using the tapping mode. The microscope was mounted with a rectangular tip working in a nominal resonant frequency of about 75 kHz. The images were processed using Nanoscope Analysis software version 1.5.

### Statistical analysis

Measurements of cells lengths were made based upon the SEM and DIC images. Statistics were calculated using the Kruskal-Wallis test in GraphPad Prism 6 software (GraphPad Software, San Diego, CA, USA). *P*-values less than 0.05 were considered statistically significant.

## Results

### Morphological characterization of differentiating forms of *T. cruzi* during metacyclogenesis in vitro

To obtain a detailed description of the surface and morphology of *T. cruzi* during metacyclogenesis, SEM analysis was performed. We focused on characterizing mainly the intermediate forms obtained during in vitro differentiation.

**Fig. 1:**
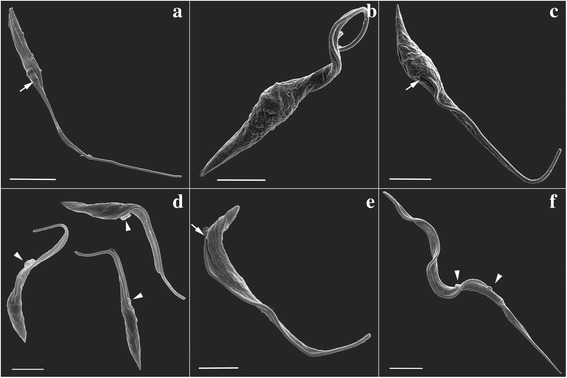
SEM analysis of *T. cruzi* during metacyclogenesis. **a** Control epimastigote not subjected to nutritional stress. The flagellum emerges from the anterior portion of the cell body (arrow). **b** Protozoan subjected to nutritional stress for 2 h. **c** Adhered cells after 48 h in differentiation medium. The flagellum emerges from the middle of the cell body (arrow). **d** Cells collected from the supernatant after 72 h in differentiation medium. Arrowheads indicate membrane shedding at the flagellar pocket. **e**, **f** Purified trypomastigotes fraction. **e** Protozoan displaying an epimastigote-like form with the flagellum emerging from the posterior end of the cell body (arrow). **f** A parasite displaying a typical metacyclic trypomastigote morphology. Arrowheads indicate membrane shedding. *Scale-bars*: **a**, 5.0 μm; **b**-**f**, 2.5 μm

SEM images revealed that, in contrast to epimastigotes (Fig. 1a), protozoans subjected to nutritional stress for 2 h displayed a twisted cell body and a wrinkled cell surface (Fig. 1b). This nutritional stress was necessary to trigger the differentiation process, and the cells were cultured further in differentiation medium. With longer culturing, these protozoans subsequently adhered to the culture flask and were released into the supernatant as metacyclic trypomastigotes. The greatest numbers of metacyclic trypomastigotes were typically obtained after culturing for 72 h. To analyze the intermediate forms, we collected adhered and non-adhered *T. cruzi* after culturing for 48 and 72 h. The adhered cells after 48 h in differentiation medium underwent a shrinkage of the cell body, and many protozoans displayed a conical-shaped morphology at their posterior end (Fig. 1c). At 48 h, the flagellum could be observed emerging from the middle of the parasite’s cell body, indicating that the repositioning of this structure had been initiated (Fig. 1c, arrow).

Protozoans collected from the supernatant after culturing for 72 h in differentiation medium displayed a smoother cell surface, an elongated morphology (Fig. 1d), and membrane shedding at the flagellar pocket region (Fig. 1d, arrowheads). One method for purifying metacyclic trypomastigotes involves performing ion chromatography using the culture supernatant. In the purified samples obtained from supernatants after 72 h of culturing, few parasites displayed an epimastigote morphology, although each of these cells already had flagellum emerging from the posterior end of their cell body (Fig. 1e, arrow). Indeed, most cells purified from these samples displayed the typical morphology of metacyclic trypomastigotes (Fig. 1f).

**Fig. 2:**
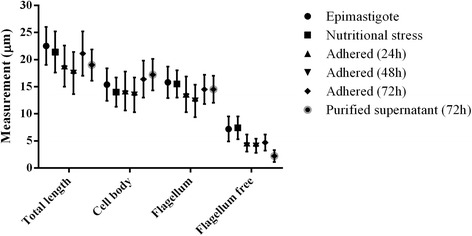
Measurements of *T. cruzi* during metacyclogenesis. Values are expressed in micrometers (μm)

SEM images were used to measure the cell bodies and flagella lengths in order to track the morphological dynamics of *T. cruzi* forms during the differentiation process. These measurements revealed that the total body length, which includes the cell body and flagellum length, decreased during differentiation. However, when the cell body length was considered separately, values increased from epimastigotes to metacyclic trypomastigotes, whereas the opposite was observed in relation to the flagellum. A statistical analysis confirmed that there was a significant difference in the total length of the epimastigotes compared to the intermediates collected at various differentiation time points and the trypomastigotes (Fig. 2). One hundred cells were measured in each stage for determine the protozoa length, the cell body and the flagellum. Cumulatively, these results suggest the existence of distinct intermediate forms during metacyclogenesis in *T. cruzi*.

### Classification and quantification of *T. cruzi* forms obtained during metacyclogenesis in vitro

**Fig. 3:**
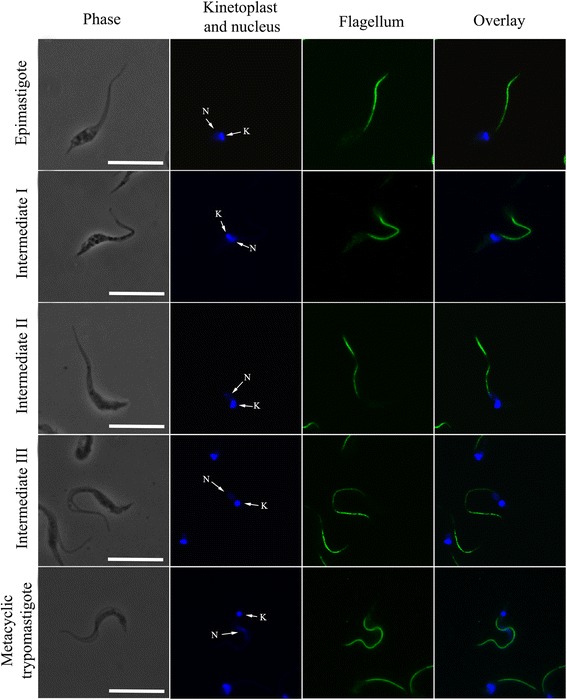
Visualization of distinct forms of *T. cruzi* during metacyclogenesis. Images of *T. cruzi* during metacyclogenesis, showing the position of the nucleus (N) and the kinetoplast (K). The nucleus is localized centrally and becomes more elongated from the epimastigote to the trypomastigote stages. The flagellum and kinetoplast are repositioned from the anterior to the posterior region of the cell body along metacyclogenesis. Five forms were observed: epimastigote, intermediate I, intermediate II, intermediate III, and trypomastigote. *Scale-bars*: 10 μm

In order to classify the intermediate forms observed by SEM, protozoans obtained at different timepoints during metacyclogenesis were incubated with DAPI, to stain the nucleus and the kinetoplast, and with a monoclonal antibody to highlight the flagellum. By examining kinetoplast morphology and its spatial relationship to the nucleus, in addition to the shape of the cell body and the region where the flagellum emerges from the cell surface, we confirmed that these parasites display distinct morphologies during differentiation. Five distinct forms were identified. First, the epimastigote, that presents an elongated shape and a kinetoplast positioned anterior to a rounded nucleus (Fig. 3, first row). Secondly, the intermediate I form consisted of cells with morphologies similar to the epimastigotes; however, the posterior part of the cell body was wider and the disk-shaped kinetoplast was located laterally to the rounded nucleus (Fig. 3, second row). Thirdly, the intermediate II form consisted of cells with a sharper body. They also displayed a slightly elongated nucleus and a disk-shaped kinetoplast located at the posterior end of the cell (Fig. 3, third row). Fourthly, the intermediate III form consisted of cells with morphologies similar to epimastigotes. However, they displayed an elongated nucleus and a globular-shaped kinetoplast located in the posterior region of the parasite (Fig. 3, fourth row). Fifthly, the metacyclic trypomastigote that presents a thinner and more sinuous body shape when compared to the other forms. A globular kinetoplast was also found to be located posterior to an elongated nucleus (Fig. 3, fifth row).

During metacyclogenesis, the width of the cell body varied with respect to the area of the nucleus (Table 1). Thirty cells in each stage were measured. The higher values observed at the intermediate I stage are related to the fact that the kinetoplast is positioned laterally to the nucleus during this stage. Conversely, the lower values observed in trypomastigotes indicates that these forms are more slender with an elongated nucleus compared to the other developmental stages analyzed.

**Table 1:** Measurements of *T. cruzi* cell body with respect to the area of the nucleus during metacyclogenesis. Values are expressed in micrometers (μm)

	Epimastigote	Intermediate I	Intermediate II	Intermediate III	Trypomastigote
Width of the cell body (in the area of the nucleus)	1.89 ± 0.31	2.21 ± 0.28	1.49 ± 0.43	1.36 ± 0.19	1.14 ± 0.18

Comparative analyses of flagellum and DAPI-stained cells also showed that the overall cell lengths of the parasites decreased during differentiation from 25.6 μm in epimastigotes to 19.4 μm in the intermediate III stage. Interestingly, the cell length increased as the parasite differentiated in the metacyclic trypomastigote stage. Protozoans at this stage displayed an average cell length of 29.6 μm. Cumulatively, this comparative analysis corroborated our previous SEM observations.

**Fig. 4:**
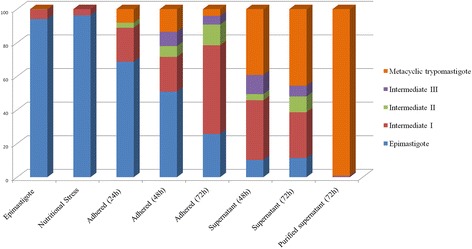
Quantification of distinct forms of *T. cruzi* during metacyclogenesis. The percentage of cells are counted at different time points during differentiation. Note that as metacyclogenesis proceeds, the number of epimastigotes and adhered parasites decreases to generate free metacyclic trypomastigotes in the supernatant. The largest number of intermediate forms are observed after 72 h of differentiation. Data were obtained from three independent experiments

In order to study the predominance of distinct forms during parasite differentiation, we quantified each form using the morphological criteria described above (Fig. 4). We observed that 24 h after the induction of metacyclogenesis, 68.7% of adhered parasites had morphological features consistent with epimastigotes, while 20.2%, 3% and 8.1% appeared to correspond to the intermediate I, intermediate II and trypomastigote forms, respectively. We did not identify cells in the intermediate III stage in this sample. Within 48 h of metacyclogenesis induction, the percentage of adhered epimastigotes decreased to 50.9%, and the amount of intermediate I and II forms was 27.1%. In the case of cells isolated from the supernatant 48 h after metacyclogenesis induction, 35.5% of the parasites were the intermediate I form and 39.16% were metacyclic trypomastigotes. The percentage of trypomastigotes increased to up to 45.6% in the supernatant 72 h after the induction of differentiation. At this point (72 h), the number of adhered epimastigotes decreased to 11.4%, and 43% of parasites corresponded to intermediate forms, most of which appeared to be in the intermediate I stage. A small percentage of trypomastigotes adhered to the substrate was observed from 24 to 72 h (< 4%). In contrast, an analysis of the supernatant revealed a decrease in the percentage of intermediate forms and an increase in the percentage of metacyclic trypomastigotes during the same period.

Cumulatively, this analysis confirmed the presence of distinct intermediate forms during the differentiation process, which were mainly observed in adhered cells samples. The intermediate I stage was the most abundant, while the intermediate II and III forms were less frequently observed.

### Ultrastructural characterization of *T. cruzi* forms during metacyclogenesis

Samples collected at different time points during the in vitro differentiation process were observed by TEM and then subjected to FIB analysis to obtain 3D reconstruction of the parasites. The goal of this was to provide a detailed description of the distinct intermediate forms classified in this work.

**Fig. 5:**
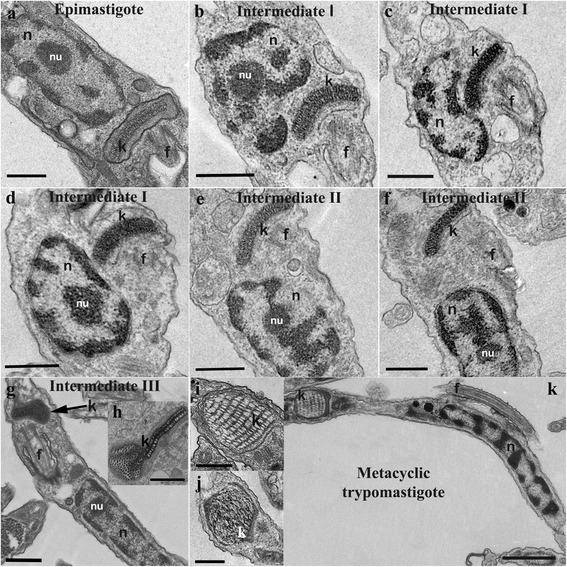
Ultrastructural analysis of *T. cruzi* during metacyclogenesis. Distinct forms are shown: **a** epimastigote, **b**-**d** intermediate I, **e**, **f** intermediate II, **g**, **h** intermediate III, and **k** trypomastigote. **a**-**k** During differentiation, the kinetoplast is repositioned from the anterior to the posterior end of the cell. **g**, **h** The kDNA undergoes topological changes during the late stages of differentiation. **i**, **j** Two types of kDNA arrangements are seen in trypomastigotes. *Scale-bars*: **a**-**g**, 0.5 μm; **h**-**j**, 0.25 μm; **k**, 0.5 μm. *Abbreviations*: f, flagellum; k, kinetoplast; n, nucleus; nu, nucleolus

**Fig. 6:**
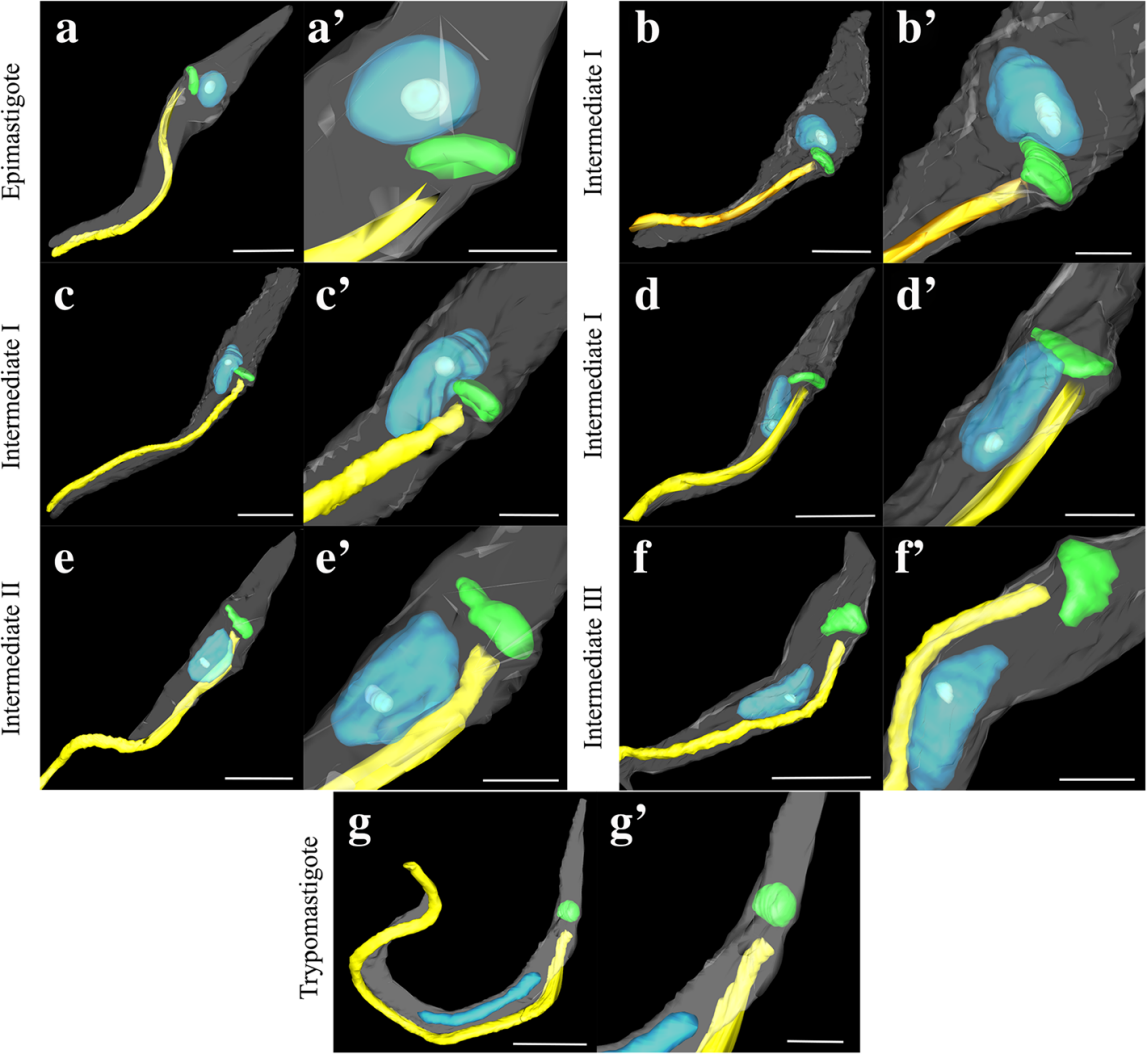
Three-dimensional reconstruction of *T. cruzi* forms during metacyclogenesis. **a** In epimastigotes, the disk-shaped kinetoplast and the flagellum can be seen in the anterior region of the parasite, and the nucleus appears rounded. **b**-**e** Repositioning of the flagellum-kinetoplast complex from the anterior to the posterior end of the parasite cell body. During kinetoplast relocation, it contacts the nucleus, which deforms and elongates. The globular kinetoplast can be observed during late stage of differentiation (**f**) and in trypomastigotes (**g**). The kinetoplast (green), nucleus (blue), flagellum (yellow), cell membrane (gray), and nucleolus (light blue) are shown. *Scale-bars*: **a**-**g**, 2.5 μm; **a’**-**g’**, 1 μm

TEM images of the intermediate forms indicated that the induction of metacyclogenesis after nutritional stress caused significant ultrastructural changes in the shape, size, and location of the protozoan kinetoplast, flagellum and nucleus (Figs. 5, 6). In epimastigotes, the flagellum and the kinetoplast were located on the protozoan anterior end and the kDNA was composed of tightly packed fibers. In the nucleus, the nucleolus was also observed, in addition to small amounts of heterochromatin located close to the nuclear envelope (Figs. 5a, 6a). After the induction of differentiation, the kinetoplast of the intermediate I form became localized laterally to the nucleus. These forms also displayed a kDNA arrangement and nuclear structure typical of epimastigotes (Figs. 5b-d, 6b-d). Furthermore, the repositioning of cellular structures within the parasite cell body compressed and deformed the nucleus (Figs. 5b-d, 6b-d). In intermediate II forms, the disk-shaped kinetoplast was localized in the posterior region of the cell (Figs. 5e, f, 6e). At this stage, we also observed parasites with densely packed kDNA similar to epimastigotes, but these parasites displayed heterochromatin spread throughout the nucleus similar to trypomastigotes. However, different from trypomastigotes, the nucleus of intermediate II cells was less elongated, and the nucleolus was still visible (Figs. 5e, f, 6e). Based on these data, we concluded that during metacyclogenesis, the kinetoplast underwent morphological changes only after it localized to the posterior end of the cell. This was also true for kDNA rearrangement. The cell morphologies observed in intermediate III were similar to those of the epimastigotes (compare Fig. 6a and f). Additionally, these parasites displayed rounded kinetoplast localized to the posterior region of the cell (Figs. 5g, 6f) and a nucleus that was not completely elongated (Fig. 6f). Interestingly, some cells possessed a chimeric kinetoplast consisting of one part that was disk-shaped and another that was globular (Fig. 5g, h). The differentiated trypomastigotes contained an elongated nucleus composed of heterochromatin that occupied most of the nuclear matrix. The nucleolus was also absent, and a globular-shaped kinetoplast was located at the posterior end of the cell (Figs. 5k, 6g). Two types of kDNA topologies were found in metacyclic trypomastigotes: one consisting of well-defined multilayers (Fig. 5i) and other consisting of an irregular arrangement without identifiable layers (Fig. 5j).

### Topological changes in kDNA during *T. cruzi* metacyclogenesis

**Fig. 7:**
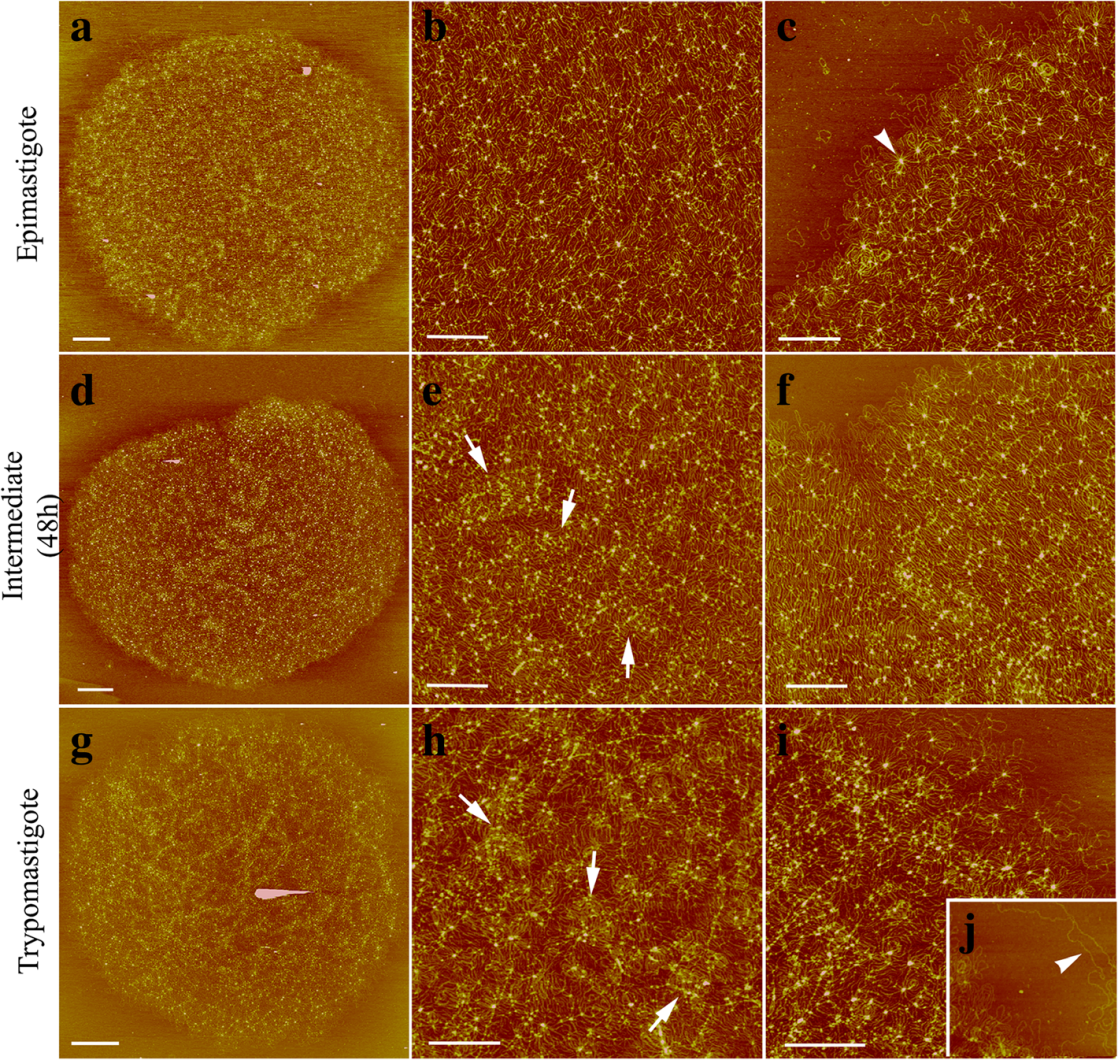
AFM of isolated kDNA networks from *T. cruzi* during metacyclogenesis. **a**, **d**, **g** Represent the entire kDNA network. **b**, **e**, **h** Show the center of the kDNA network. **c**, **f**, **i**, **j** Correspond to the outer edges of the kDNA network. The kDNA is composed of a massive network of interlocked DNA molecules. It is most uniformly distributed in epimastigotes (**a**-**c**). This uniformity decreases in the intermediate forms (**d**-**f**) and it is even lower in the trypomastigotes (**g**-**i**). In these last two forms, several foci containing a high concentration of kDNA fibrils are shown (**e**, **h**, arrows). **j** Part of a maxicircle is shown in the trypomastigotes (arrowhead). *Scale-bars*: **a**, **g**, 1 μm; **d** 2 μm; **b**, **c**, **e**, **f**, **h**, **j**, 0.5 μm

To investigate the topology of kDNA and how the kDNA network is altered during metacyclogenesis in *T. cruzi*, kDNA networks were isolated from different forms (epimastigotes, adhered parasites after 48 h of differentiation, and trypomastigotes). These samples were analyzed by AFM (Fig. 7). This revealed that networks are composed of interlocked DNA molecules that form several rosette-like structures (Fig. 7c, arrowhead). These comparative analyses highlighted interesting differences in the topologies of kDNA networks among distinct *T. cruzi* forms. In epimastigotes, the network (Fig. 7a-c) displayed a more uniform distribution of kDNA molecules when compared to both the intermediate and trypomastigote stages. For the intermediate forms (Fig. 7d-f), kDNA fibers were more concentrated in certain regions of the network (Fig. 7e, arrows), and this was even more obvious in the kDNA networks of trypomastigotes (Fig. 7h, arrows). We also observed part of a maxicircle on the kDNA network in the trypomastigotes (Fig. 7j, arrowhead). The isolated networks measured 9.54 × 8.62 μm ± 1.09 × 1.2 μm in epimastigotes, 8.12 × 7.36 μm ± 0.68 × 0.68 μm in intermediate forms, and 7.48 × 6.93 μm ± 0.66 × 0.58 μm in trypomastigotes. Fifty networks in each stage were measured. AFM analysis was essential for providing high-resolution images of alterations in the kDNA network during differentiation. As a result, we confirmed that distinct kDNA topologies exist by comparing differentiated developmental stages and intermediate forms of the parasite. It can be useful as a model to improve our understanding of how the topological arrangement of kDNA changes during metacyclogenesis.

## Discussion

During evolution, parasites are likewise subjected to constant selective pressures that make them better adapted to certain environments. Typically, protozoa respond to stress conditions through cell differentiation, which occurs in many trypanosomatids [2, 25]. *Trypanosoma cruzi* is a heteroxenic parasite that alternates its life-cycle between an insect host and mammalian cells. Within the insect’s gut, proliferative and nonpathogenic epimastigotes differentiate into trypomastigotes, which are the non-proliferative, infective forms. This differentiation process is known as metacyclogenesis. During metacyclogenesis, the protozoan takes on different forms as a result of changes in gene expression that lead to morphological, ultrastructural, metabolic, and physiological modifications [11, 16, 26]. Kollien & Schaub [3] identified different morphological stages of *T. cruzi* in the rectum of *Triatoma infestans*; however, most of them, especially the intermediate ones, have not been fully characterized from a structural point of view or even named.

**Fig. 8:**
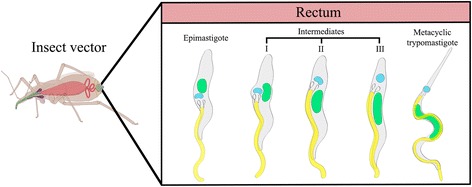
Summary of *T. cruzi* differentiation in the intestine of the insect vector during metacyclogenesis. This model is based on the forms observed in this study using an in vitro differentiation assay. These forms are compatible with those previously identified in the insect rectum. Schematic representation courtesy of Jean Oliveira Santos

In this work, we identified different intermediate stages of *T. cruzi* using an in vitro metacyclogenesis system and methods for morphological and ultrastructural analyses. As result of a temporal description of this differentiation process, we identified and characterized three distinct intermediate forms that were named as intermediates I, II and III. Intermediate I corresponds to cells in which the flagellum and disk-shaped kinetoplast are repositioned from the anterior to the posterior region of the cell body with a localization lateral to the nucleus. During the reorganization of these cellular structures, the kinetoplast contacts the nucleus, which deforms and becomes more elongated. This observation agrees with previous reports [11, 17]. An important finding of the present work was the demonstration that the repositioning of the flagellum and kinetoplast and the elongation of the nucleus are early events in the differentiation process. They precede changes in the shape of the kinetoplast and the topological rearrangement of the kDNA. Interestingly, soon after its localization to the posterior end of the cell, the kinetoplast still appears disk-shaped and contains densely packed kDNA. This is characteristic of the intermediate II stage, which also displays a slightly elongated nucleus and morphology similar to the epimastigote. In the intermediate III stage, this epimastigote-like shape is maintained, but the nucleus is more elongated. The flagellum also emerges from the posterior region of the cell where the kinetoplast is localized. Additionally, the kinetoplast takes on a globular appearance with a looser arrangement of kDNA (Fig. 8 summarizes this description).

Previous studies proposed that the changes in the 3D organization of the cytoskeleton lead to the repositioning of cellular structures within the cell bodies of trypanosomatids [27–29]. While studying the arrangement of microtubules during the intracellular cycle of *T. cruzi*, Meyer & De Souza [27] proposed that in amastigotes, the increased length of the flagellar microtubules and microtubules assembled around the kinetoplast causes the elongation of the parasite cell body. This helps shape it into the long and slender form characteristic of the trypomastigote. Furthermore, since this microtubule elongation occurs in the direction of flagellum growth, the repositioning of the flagellum-kinetoplast complex towards the posterior end of the parasite would probably occur passively, and not as a result of the migration of the basal body and kinetoplast. Previous works demonstrated that the flagellar attachment zone is a key structure regulating the overall length of the cell and organelle positioning, and it is likely that the wide range of trypanosomatid morphologies results from modulation of FAZ expression [28–30]. Therefore, it is possible that the modifications in the cell shape and organelle positioning observed in intermediate stages of metacyclogenesis in *T. cruzi* result from the cytoskeletal rearrangements.

Ferreira et al. [17] previously proposed that *T. cruzi* exhibits three intermediate forms: Ia, Ib and Ic. These are defined according to the positioning of the kinetoplast with respect to the nucleus. In intermediate Ia, the kinetoplast displacement is just beginning and this structure is still observed in the anterior portion of the cell body. In Ib form, the kinetoplast becomes positioned lateral to the nucleus. Finally, in stage Ic, the kinetoplast shifts past the nucleus to the posterior region of the cell body. However, in this study, the authors did not consider changes in the kinetoplast morphology or the shape of the cell body during differentiation. They also did not examine alterations in kDNA topology. In fact, the Ia, Ib and Ic intermediates correspond to the parasite form in which kinetoplast repositioning occurs, regardless of whether this structure is located anterior, side-by-side, or posterior to the nucleus. Thus, in order to simplify this nomenclature system, we called all these forms (Ia, Ib, and Ic) “intermediate I.” The intermediate I was initially referred to as “early intermediate” by Kleffman et al. [7], and the intermediate II, which was not described by Ferreira et al. [17], was termed “late intermediate” by the same authors. Considering previously published data and our new findings, we propose that the early and late intermediate forms should be renamed intermediate I and II, respectively. We also suggest that the intermediate III should also be considered in this new classification system for *T. cruzi* metacyclogenesis. This last form was originally identified by Kollien & Schaub [3], but never named. Cumulatively, our revised naming system more accurately represents the complexity of the intermediate forms of *T. cruzi* as it undergoes metacyclogenesis in the small intestine and rectum of the insect *T. infestans*.

After characterizing epimastigotes, trypomastigotes, and the intermediate forms, we offer a temporal and more complete description of *T. cruzi* differentiation during metacyclogenesis. Specifically, we observed that 24 h after nutritional stress, almost 70% of the epimastigotes adhered to the substrate, supporting the hypothesis that the parasite-substrate adhesion interaction is essential for differentiation [7, 11]. Our results also showed that, as differentiation progressed, the percentage of adhered epimastigotes decreased. Meanwhile, the amount of adhered intermediates increased greatly from 48 to 72 h. During the same period, the percentage of intermediate forms and trypomastigotes released into the supernatant increased markedly. Only a small percentage of intermediate III and trypomastigotes remained adhered to the substrate after 72 h in the differentiation medium. This suggests that most trypomastigotes obtained by in vitro metacyclogenesis originated from cells in intermediate stages that had already detached from the substrate. Only a small number of parasites completed differentiation while still attached to the substrate. Previous studies already demonstrated that both epimastigotes and trypomastigotes remain attached to the insect rectum wall. However, trypomastigotes and their intermediate stages have a higher detachment rate compared to epimastigotes [31, 32]. This phenomenon likely results from changes in the expression and/or localization of cell-surface molecules in these parasites, as proposed by Bonaldo et al. [11]. According to these authors, during the epimastigote-to-trypomastigote transformation, a decrease of cell surface components involved in substrate adhesion occurs, resulting in the release of trypomastigotes into the supernatant as they complete differentiation. This explains, at least in part, the observed increase in the number of intermediate forms and trypomastigotes in the supernatant in our study. The morphogenetic transformation during metacyclogenesis in *T. cruzi* is related to the control of gene expression, through polysomal mobilization and protein posttranslational modifications [12, 13]. Proteomic analyses showed that differences between epimastigotes and trypomastigotes involve essential processes, such as energy metabolism, catabolism, proteolysis, and the structural organization. In addition, metacyclic trypomastigotes presented a higher expression of proteins that enhances parasite survival and virulence [13].

Our SEM analysis showed that as soon as the differentiation process is triggered, the parasite undergoes a slight shrinkage and twisting of the cell body, which is accompanied by a wrinkling of the cell surface. Likewise, after exposing epimastigotes to hyperosmotic stress by incubation with sorbitol, it has been shown that the cells display similar surface shrinkage in a process involving water transport by aquaporins to the contractile vacuole [33]. In nature, *T. cruzi* is subjected to changing osmotic conditions. For example, in the intestine and the rectum of the insect vector where differentiation occurs, a higher osmolarity is observed compared to the stomach [3]. In vitro, during metacyclogenesis, it was found that the parasites suffer osmotic stress when transferred to the TAU medium, which is slightly hypertonic compared to the LIT medium [10]. Similarly, in axenic cultures, an increase in osmolarity was found to induce the transformation of epimastigotes into intermediate forms, suggesting that hyperosmotic stress is part of an adaptive process culminating in cell differentiation [34].

In this study, we also observed membrane shedding during *T. cruzi* metacyclogenesis in both advanced stages of differentiation and in fully differentiated cells. Some studies have reported that *T. cruzi* epimastigotes and trypomastigotes release large vesicles from the plasma membrane, in addition to smaller vesicles that bud within the flagellar pocket [35–39]. Recently, it was demonstrated that these shed vesicles not only facilitate or significantly enhance metacyclogenesis in *T. cruzi*, but they also increase the susceptibility of mammalian cells to infection [36]. Taken together, these data indicate that the shedding of vesicles in *T. cruzi* represents an essential means by which the parasite infects hosts and survives.

Our structural analyses revealed that changes in the shape of the kinetoplast and the arrangement of kDNA only occur after the complete repositioning of these structures to the posterior region of the cell. In fact, rounded kinetoplast was never seen in the anterior region of the parasites or during kinetoplast displacement. It was interesting to observe that in intermediate III stage cells, the kinetoplast sometimes displayed chimeric features. Part of the kinetoplast had a disk-shaped appearance while the other part had a globular shape. This suggests that the topological rearrangement of kDNA begins at one extremity of the structure. Since it was not often that we observed this chimeric morphology, we assume that this occurs very briefly during the transition between intermediate stages II and III.

In order to characterize kDNA rearrangements during *T. cruzi* metacyclogenesis, isolated networks were analyzed by AFM. This technique has previously allowed the investigation of kDNA organization with high resolution [24, 40]. Our results showed that the size of the kDNA network decreases during metacyclogenesis, which might be related to the increased number of foci containing a higher concentration of kDNA fibrils. These were visualized particularly in trypomastigotes, but also in intermediate forms as well. In contrast, epimastigotes displayed a uniform distribution of kDNA. This suggests that the arrangement of kDNA undergoes compaction during metacyclogenesis. This may be the result of a generalized mechanism for RNA transcriptional repression in the kinetoplast [4]. However, this hypothesis requires further investigation.

In contrast to the kinetoplast, the nucleus was observed to undergo shape changes at the beginning of differentiation when the kinetoplast initiated its repositioning towards the posterior region of the cell. Previous studies demonstrated that during repositioning, the kinetoplast compresses the nucleus, which causes it to become elongated in the intermediate stages of *T. cruzi* differentiation [11, 16, 17].

In *T. cruzi,* we found that the differentiation of epimastigotes into metacyclic trypomastigotes is accompanied by nuclear reorganization. Additionally, changes in chromatin compaction were observed during metacyclogenesis, as well as a decrease in the transcriptional activity of RNA polymerase I and II [41]. Ferreira et al. [17] showed that the nucleolus is present during the intermediate I stage, which occurs at the beginning of metacyclogenesis. In our present work, ultrastructural analyses revealed that the nucleolus is present even in advanced stages of *T. cruzi* differentiation, since this nuclear domain was observed in intermediate II and III stages.

Traditional TEM provides useful data about the cellular ultrastructure of trypanosomatids, including the organization of its organelles. However, this method is limited by the area of the ultrathin sections, thus generating bi-dimensional images. In contrast, FIB-SEM is a powerful new tool for biological research. Using FIB-SEM, a preselected region of interest from a sample embedded in resin is milled off with an ion beam and then imaged with an electron beam. By repeating this process several times, a set of data containing various planes of the object under investigation is obtained, thus furnishing a 3D reconstruction of the organelles and structures of the cells [42]. After obtaining a 3D model of different intermediate forms of *T. cruzi* during metacyclogenesis*,* we could confirm that the nucleus is deformed during the repositioning of the kinetoplast and flagellum. We also confirmed that the nucleolus disappears in trypomastigotes. Recent data obtained by FIB-SEM showed that during the late stages of *T. cruzi* differentiation, the Golgi complex translocates to the posterior end of the cell body and the cytostome-cytopharynx complex disappears. Furthermore, this same study raised the question of whether reservosomes disappear or are simply remodeled during metacyclogenesis, originating the protease-containing compartments present in typomastigotes [43].

## Conclusions

Trypanosomatids possess fascinating biological characteristics and represent a very useful model for studying a wide variety of cellular, molecular, and evolutionary processes. Metacyclogenesis is an adaptive differentiation that enables parasites to survive in diverse environments [2]. In this study, we examined *T. cruzi* metacyclogenesis in vitro*,* which enabled us to perform a temporal description of the distinct forms that arises during parasite differentiation. Based upon our results and the data available in the literature, we propose a unified nomenclature for the intermediate forms of this microorganism, also considering the differentiation of epimastigote into trypomastigote in the intestine of the insect vector (Fig. 8). This new classification may simplify future analyses using biochemical and molecular approaches, including those that investigate the kDNA reorganization during metacyclogenesis. Ultimately, we hope that this work provides a useful framework for studying the biology of a protozoan that poses a significant threat to public health worldwide.

## Abbreviations

AFM: atomic force microscopy
DAPI: 4′,6-diamidino-2-phenylindole
DEAE-cellulose: 2-(diethylamino) ethyl ether celulose
DIC: differential interference contrast
DTU: discrete typing units
EDTA: ethylenediaminetetraacetic acid
FAZ: flagellar attachment zone
FIB-SEM: focused ion beam scanning electron microscopy
kDNA: kinetoplast DNA
LIT: liver infusion tryptose
SDS: sodium dodecyl sulfate
SEM: scanning electron microscopy
TAU: triatomine artificial urine
TEM: transmission electron microscopy
